# The Epigenetic Factor CBP Is Required for the Differentiation and Function of Medial Ganglionic Eminence-Derived Interneurons

**DOI:** 10.1007/s12035-018-1382-4

**Published:** 2018-10-17

**Authors:** Alejandro Medrano-Fernández, Jose M. Delgado-Garcia, Beatriz del Blanco, Marián Llinares, Raudel Sánchez-Campusano, Román Olivares, Agnès Gruart, Angel Barco

**Affiliations:** 1 Instituto de Neurociencias (Universidad Miguel Hernández - Consejo Superior de Investigaciones Científicas), Av. Santiago Ramón y Cajal s/n. Sant Joan d’Alacant. 03550, Alicante, Spain; 2 Division of Neurosciences, Pablo de Olavide University, 41013 Seville, Spain

**Keywords:** Interneurons, CBP, Rubinstein-Taybi syndrome, Epilepsy, Intellectual disability, Neuroprogenitor

## Abstract

The development of inhibitory circuits depends on the action of a network of transcription factors and epigenetic regulators that are critical for interneuron specification and differentiation. Although the identity of many of these transcription factors is well established, much less is known about the specific contribution of the chromatin-modifying enzymes that sculpt the interneuron epigenome. Here, we generated a mouse model in which the lysine acetyltransferase CBP is specifically removed from neural progenitors at the median ganglionic eminence (MGE), the structure where the most abundant types of cortical interneurons are born. Ablation of CBP interfered with the development of MGE-derived interneurons in both sexes, causing a reduction in the number of functionally mature interneurons in the adult forebrain. Genetic fate mapping experiments not only demonstrated that CBP ablation impacts on different interneuron classes, but also unveiled a compensatory increment of interneurons that escaped recombination and cushion the excitatory-inhibitory imbalance. Consistent with having a reduced number of interneurons, CBP-deficient mice exhibited a high incidence of spontaneous epileptic seizures, and alterations in brain rhythms and enhanced low gamma activity during status epilepticus. These perturbations led to abnormal behavior including hyperlocomotion, increased anxiety and cognitive impairments. Overall, our study demonstrates that CBP is essential for interneuron development and the proper functioning of inhibitory circuitry in vivo.

## Introduction

The multiple actions of the mammalian brain depend on the precise spatiotemporal control of synaptic communication within local and long-range neuronal networks. Interneurons are γ-aminobutyric acid-containing (GABAergic) neurons that oversee inhibitory activity within the brain. Although fewer in number than excitatory neurons, inhibitory neurons play a crucial role in fine-tuning neuronal firing and shaping the activity and rhythms of neuronal circuits [1, 2]. They constitute a highly heterogeneous group of cells with different morphological, neurochemical, and electrophysiological properties. Interestingly, the different classes of interneurons arise from anatomically restricted niches in the subpallium, at the base of the embryonic telencephalon. They first undergo tangential migration to invade the olfactory bulb and cortical structures, and later radial migration to reach their specific locations [2, 3]. The median ganglionic eminence (MGE) is one of the main birthplaces for the most abundant interneuron classes in the brain, those expressing parvalbumin (PV) and those expressing somatostatin (SST). These cells are critical for network activity dynamics and stability, as well as for the orchestration of brain oscillations. As a result, disrupting their development may lead to severe neurological conditions, including epilepsy and cognitive deficiencies [4, 5].

During brain development, the production of excitatory and inhibitory neurons is precisely regulated. In particular, the development of inhibitory circuits relies on a network of transcription factors (TF) that control the sequential and region-specific activation of complex gene programs [2, 3, 6, 7]. For example, the TF Nkx2-1, which is specifically expressed in neural progenitors of the MGE [8], and the homeobox TFs Dlx1 and Dlx2, which are expressed by progenitors within the ventral telencephalon [9], are all important for promoting interneuron differentiation. While some of these TFs act as docking sites for chromatin remodeling enzymes, the direct contribution of such epigenetic factors remains largely unexplored. Among the candidate chromatin-modifying enzymes to play a role in interneuron differentiation, we decided to focus on the CREB-binding protein (CBP *aka* KAT3A), a lysine acetyltransferase (KAT) and transcriptional co-activator. This epigenetic enzyme adds acetyl groups to lysine residues of numerous nuclear proteins, including the four nucleosome histones [10–12]. In support of CBP having a specific role in interneuron function and differentiation, it was recently shown that interneuron development is transiently compromised in CBP heterozygous mice [13]. Consistent with this finding, ex vivo experiments also indicated that knocking down CBP in cultured MGE precursors interfered with interneuron morphogenesis [13]. However, the role of CBP in vivo and in other interneuron subtypes remains unexplored.

To investigate the role of CBP in interneuron development in vivo, we generated mice in which CBP was specifically removed from the interneuron progenitors of the MGE. Our results demonstrate that CBP is critical for the proper integration of different classes of MGE-born interneurons into cortical and hippocampal circuits. Furthermore, interfering with this process leads to a number of physiological and behavioral abnormalities including the emergence of spontaneous seizures and compromised brain oscillations and plasticity, all of which result in cognitive impairments.

## Materials and Methods

### Animals

The generation of *Crebbp*^*f/f*^ [14], Nkx2.1-cre [15], and CMV-fxSTOP-tdTomato [16] mice has been described previously. The last two strains are available at the Jackson laboratory with the stock numbers #8661 and #7914, respectively. The *Crebbp* floxed strain was provided by Beat Lutz (Institute of Molecular Biology, Mainz, Germany). The genetic background of all mice is C57BL/6J. Experiments were conducted blind, and genotypes were provided for statistical analyses. Both male and female mice were examined. Every animal was used in a single experiment except when it is indicated otherwise. Nkx2.1-CBP_KO_ (pNkx2.1-cre::*Crebbp*^f/f^) were compared with their control littermates (*Crebbp*^f/f^, with no cre recombinase expression), and Nkx2.1-CBP_KO_/tdTOM (pNkx2.1-cre::*Crebbp*^f/f^::CMV-fxSTOP-tdTomato) were compared with Nkx2.1-tdTOM controls (pNkx2.1-cre::CMV-fxSTOP-tdTomato). For genotyping, we used the following primers: *CBPfloxed* allele fw: 5’-CCTCTGAAGGAGAAACAAGCA and rv: 5’-ACCATCATTCATCAGTGGACT (wild-type band: 230 bp; mutant band: 300 bp); *Cre* recombinase transgene fw: 5’-AGATGTTCGCGATTATC-3′ and rv: 5’-AGCTACACCAGAGACGG-3′ (490 bp amplicon). All mice were maintained and bred under standard conditions, consistent with Spanish (BOE 34/11370-421, 2013) and European Union Council (2010/63/EU) regulations and approved by the Institutional Animal Care and Use Committee.

### Histology

**Fig. 1:**
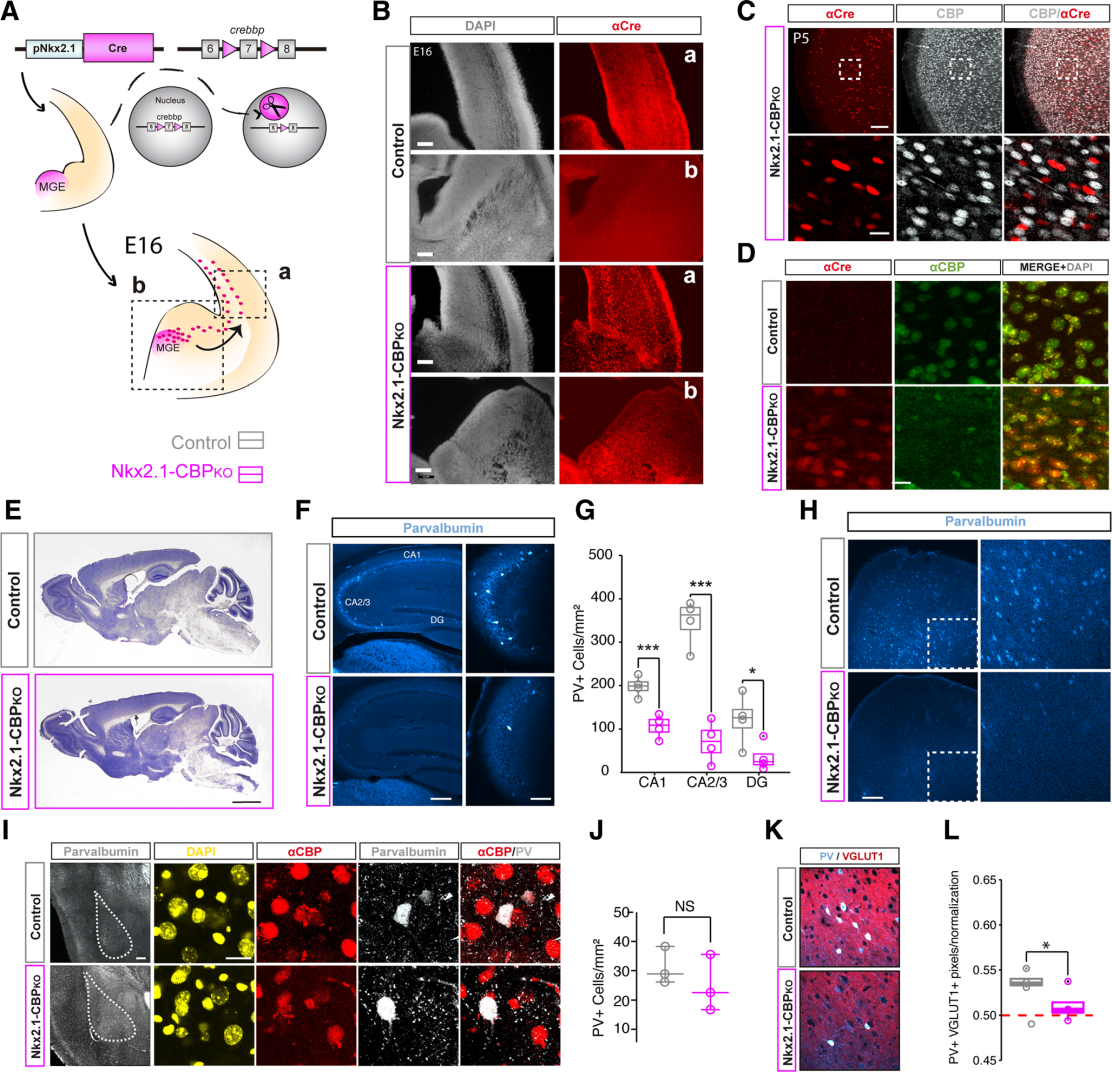
Nkx2.1-CBP_KO_ mice have a reduced number of PV+ interneurons. **A** Scheme of genetic manipulation. The *Nkx2-1* promoter drives the expression of Cre recombinase in MGE interneuron progenitors of *Crebbp*^*f/f*^ mice, resulting in the elimination of exon 7 of *Crebbp* in these cells. **B** The expression of Cre-recombinase is detected in the expected areas in Nkx2.1-CBP_KO_ E16 embryos Scale bars: 100 μm (**a**), 200 μm (**b**). **C** Double immunostaining for Cre recombinase and CBP in the parietal cortex of P5 mice. The detection of Cre recombinase coincides with the loss of CBP immunoreactivity. Scale bars: 100 μm (left), 20 μm (right). **D** Double immunostaining for Cre recombinase and CBP in striatal tissue and area that contains Nkx2-1-expressing cells in adult mice. Scale bar: 10 μm. **E** Representative images of Nissl staining in brain slices of Nkx2.1-CBP_KO_ and control littermates. Scale bar: 1 mm. **F** Representative images of immunostaining with an anti-PV antibody. Scale bars: 200 μm (left) and 10 μm (right). **G** Quantification of the number of PV+ cells in the different hippocampal subfields. Nkx2.1-CBP_KO_ mice have fewer PV+ cells in all substructures (DG, t_6_ = 2.55, *p* = 0.04; CA1, t_6_ = 5.17, *p* = 0.01; CA3, t_6_ = 7.63, *p* = 0.001). **H** Cortical areas, such as the frontal pole, also show a lower number of PV+ cells in Nkx2.1-CBP_KO_. Scale bar: 200 μm. **I** Immunostaining with an anti-PV antibody in the amygdala. Scale bars: 200 μm (left) and 20 μm (right). **J** The quantification of the number of PV+ cells in the amygdala revealed no significant difference. **K** Representative images of co-immunostaining with anti-PV and anti-VGlut1 antibodies. Scale bar: 50 μm. **L** Quantification of P*V*/VGLUT1 co-localization indicates that the glutamatergic innervation of PV+ interneurons is reduced in Nkx2.1-CBP_KO_ (t_6_ = 2.62, *p* = 0.04, unpaired *t* test). *P < 0.05; ***P < 0.001

**Fig. 2:**
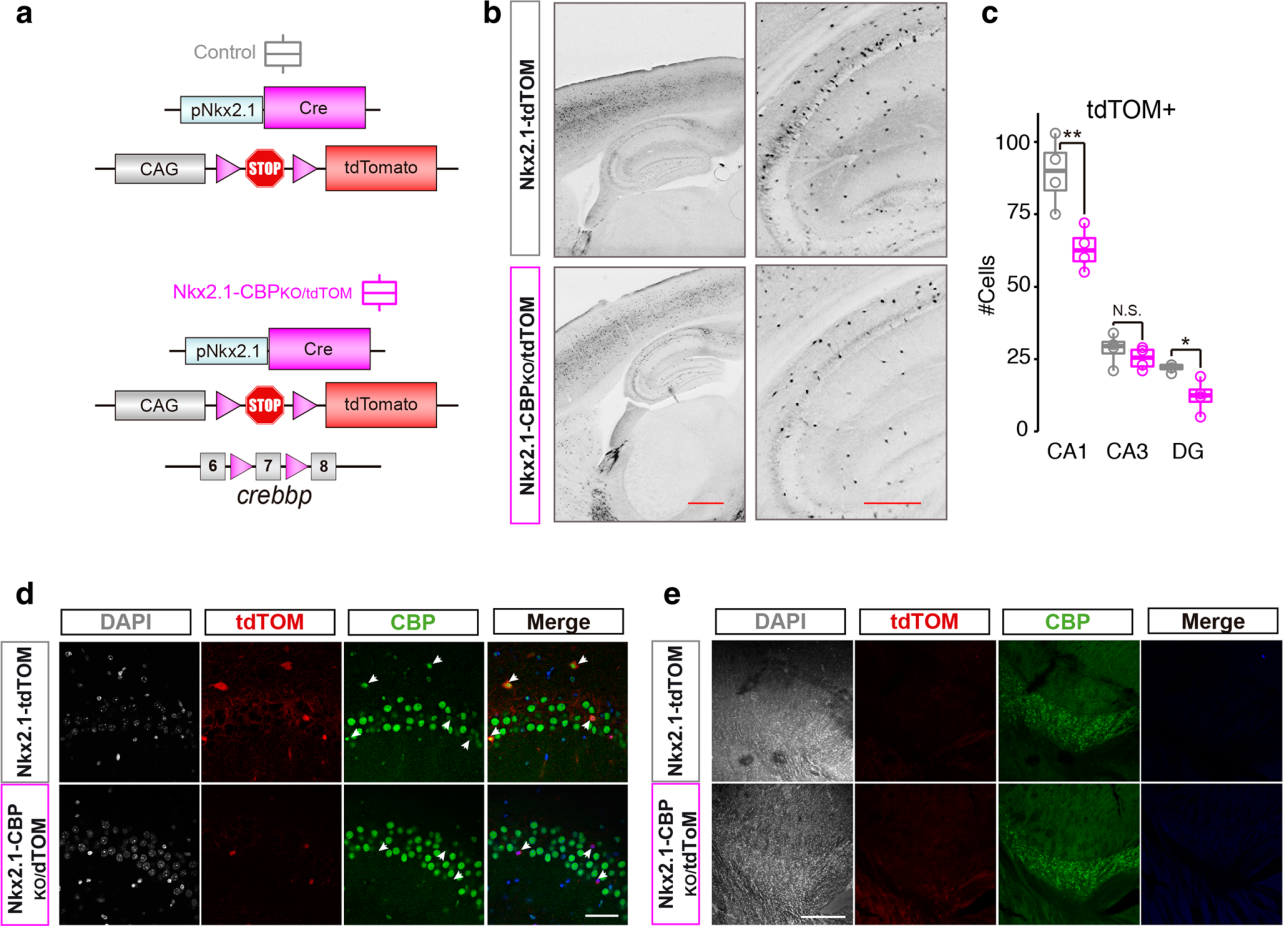
Genetic mapping demonstrates that Nkx2.1-CBP_KO_ mice have fewer MGE-derived cells. **a** Scheme of the genetic mapping strategy. The *Nkx2-1* promoter drives the expression of the Cre recombinase at MGE progenitors leading to the removal of a STOP cassette that otherwise prevents transcription of the red fluorescent protein tdTOM. **b** Representative images showing the dramatic reduction in the number of tdTOM+ cells in Nkx2.1-CBP_KO_/tdTOM mice. Scale bars: 500 μm. **c** Experimental mice have fewer tdTOM+ cells in CA1(t_6_ = − 3.8, *p* = 0.01, unpaired *t* test) and DG (t_3,36_ = − 3.30, *p* = 0.04, Welch *t* test) subdivisions of the hippocampus, but not in CA3 (t_6_ = − 0.97, *p* = 0.36, unpaired *t* test). **d** tdTOM+ cells in Nkx2.1-CBP_KO_/tdTOM do not express CBP. We present images of the CA1 subfield, but similar results were observed in other areas. Scale bar: 50 μm. **e** No differences are observed in the reticular nucleus of the thalamus. Scale bar: 200 μm. *P < 0.05; **P < 0.01

**Fig. 3:**
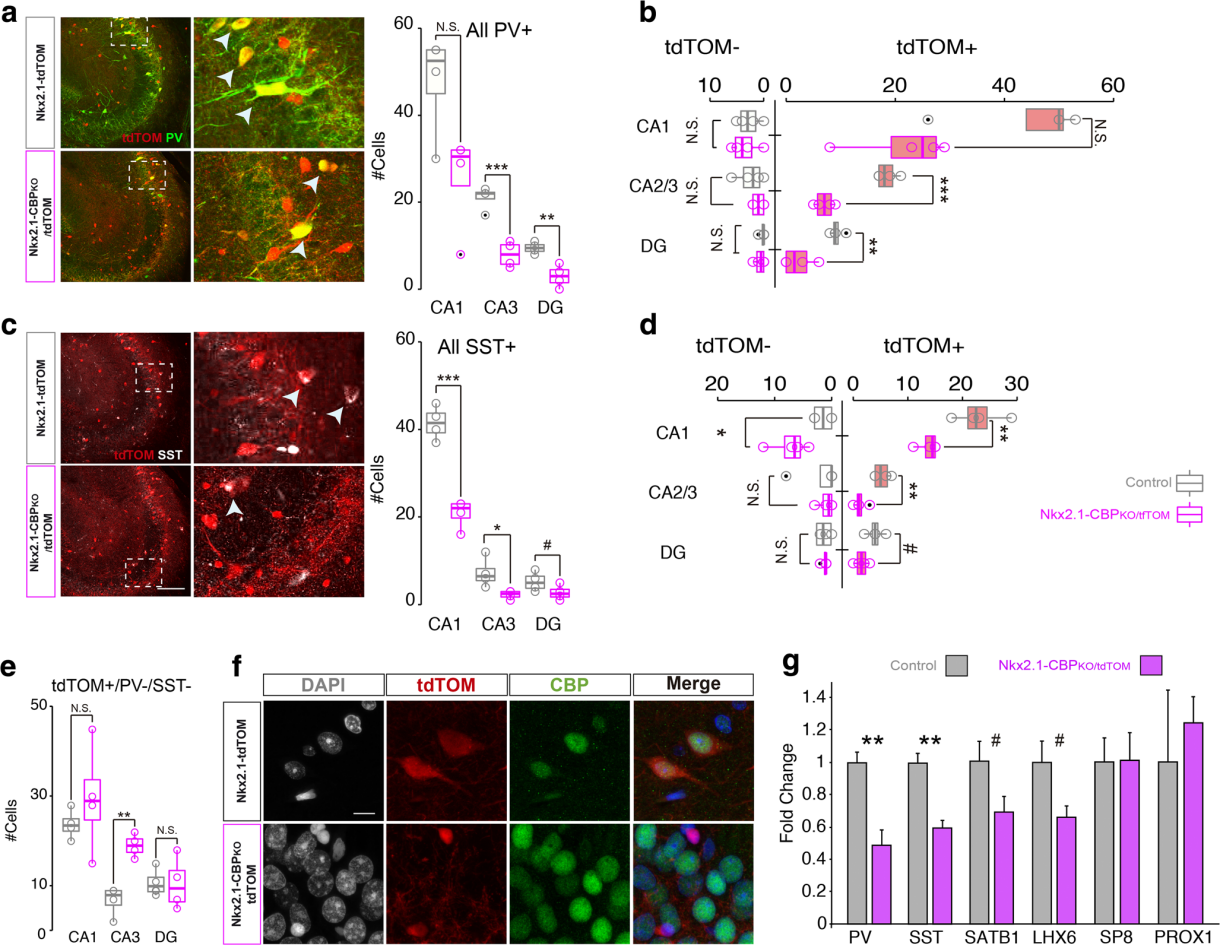
Subtype-specific characterization of MGE-derived interneurons. **a** Representative image of the hippocampus employed for quantification of PV+ cells (left) and quantification of all PV+ cells regardless of tdTOM expression (right). **b** Separate quantification of tdTOM positive and negative PV+ cells. PV+ cells that are not derived from the MGE (i.e., PV+, tdTOM-) are unaffected in CBP-deficient mice (CA1, t_6_ = 0.43, *p* = 0.68, unpaired *t* test, *d* = 0.31; CA3, U = 5, *p* = 0.47, Mann-Whitney *U*, *A* = 0.31; DG, *U* = 10.5, *p* = 0.51, Mann-Whitney *U*, *A* = 0.66). However, MGE-derived PV+ interneurons (PV+, tdTOM+) were diminished in Nkx2.1-CBP_KO_/tdTOM mice (CA1, U = 2, *p* = 0.11, Mann-Whitney *U*, *A* = 0.13; CA3, t_6_ = − 8.69, *p* = 0.001, unpaired *t* test, *d* = − 6.15; DG, t_6_ = − 4.46, *p* = 0.004, paired *t* test, d = − 6.15). **c** Representative images for quantification of SST+ cells (left, same fields as in panel A) and quantification of all SST+ cells regardless of tdTOM expression (right). Scale bar: 100 μm. **d** Separate quantification of tdTOM positive and negative SST+ cells. We did not find differences in SST+ cells born outside the MGE (i.e., SST+, tdTOM-) at the CA3 (*U* = 7, *p* = 0.87. Mann-Whitney *U*, *A* = 0.44) and DG (t_6_ = − 0.07, *p* value = 0.94, unpaired *t* test, *d* = 0.05) subfields of CBP-deficient mice. However, the CA1 subfield shows a significantly larger number of SST+ cells (*U* = 16, *p* = 0.02. Mann-Whitney *U*, *A* = 1). **e** Quantification of the number of tdTOM+ cells that do not express the proteins PV or SST proteins in Nkx2.1-CBP_KO_/tdTOM and Nkx2.1-tdTOM mice. There is a statistically significant difference in the CA3 subfield (t_6_ = − 5.84, *p* value = 0.001, unpaired *t* test, *d* = − 4.13). **f** Representative images of tdTOM+/PV-/SST- cells in the CA3 subfield of Nkx2.1-CBP_KO_/tdTOM and Nkx2.1-tdTOM mice. Scale bar: 10 μm. **g** RT-qPCR assays for the indicated genes using total RNA isolated from the hippocampus of Nkx2.1-CBP_KO_ and control littermates. Statistical differences were found for *Pvalb* (PV) (t_5_ = 3.95, *p* = 0.01, unpaired *t* test) and *Sst* (SST) (t_5_ = 5.49, *p* = 0.01, unpaired *t* test) expression, as well as a clear trend in *Satb1* (SATB1) (t_5_ = 2.05, *p* = 0.09, unpaired *t* test) and *Lhx6* (LHX6) (t_5_ = 2.34, *p* = 0.07, unpaired *t* test). We observed in these four genes large effects size. #*P* < 0.1; *P < 0.05; **P < 0.01; ***P < 0.001

Histology experiments used adult mice of both sexes. The immunostainings described in Fig. 1 were conducted in 7–9-month-old mice (Nkx2.1-CBP_KO_: *n* = 4, 2 males and 2 females; control littermates: *n* = 4, 2 males and 2 females), whereas the experiments described in Figs. 2 and 3 were conducted in 2–3-month-old mice (Nkx2.1-CBP_KO/tdTOM_: *n* = 4, 2 males and 2 females; control littermates: *n* = 4, 1 male and 3 females). Statistical analysis did not reveal significant differences between sexes. Mice were perfused intracardially with 4% PFA. Sections were washed with PBS containing 0.25% Triton X-100 (PBS-T) for 5 min at room temperature. Nissl staining was performed by incubating 50-μm thick brain slices in cresyl violet for 30 min, dipping in dH2O briefly, washing in a solution of 0.1% acetate in 95% ethanol, then washing in 95% ethanol, afterwards in 100% ethanol, and last dipped in xylene twice for 3 min and rapidly mounted with Neo-Mount. For immunostaining, sections were incubated for 30 min in 3% normal calf serum (NCS) diluted with PBS-T (200 μl/well). Then, sections were incubated in the primary antibody solution (2% normal calf serum (NCS) with the antibody of interest diluted 1:500 with PBS-T to a final volume of 200 μl/well) overnight at 4 °C, and the secondary antibody incubation was performed at room temperature during 2 h. Finally, nuclei were counterstained with a 1 nM DAPI solution (Invitrogen), washed with PBS-T and PBS and later on mounted in slides with fluoromount mounting medium. The primary antibodies used in this study are α-CBP (Santa Cruz, sc-7300), α-VGLUT (Synaptic Systems, 135–304), α-GFAP (Sigma, G9269), α-parvalbumin (Swant, PV235 and Sigma, P3008), and α-Cre (gift from Schütz’s lab). For cell quantification, cells were manually counted using *ImageJ* cell counter. For each mouse, we analyzed a 50-μm deep hyperstack mosaic corresponding to hippocampal slices. Images were obtained with an Olympus confocal microscope (× 20 objective). Blind quantification of cells in the *stratum oriens* of the CA1 and CA3 subfield, the granular layer of the DG and the amygdala were conducted using images collecting the maximum fluorescence of the hyperstack. DAPI counterstaining was used as a reference for calculating the total number of cells. For VGLUT + PV+ fluorescence quantification, the numbers of pixels with signals in the two channels were quantified and divided by the amount of co-localizing pixels with one transposed channel in order to correct for the different number of cells present.

### Behavioral Analyses

A large cohort of Nkx2.1-CBP_KO_ (*n* = 16, 9 males and 7 females) and control (*n* = 17, 8 males and 9 females) littermates of 4–6 months of age was used for behavioral testing. We conducted the following tests:

#### Open Field (OF)

Mice were placed in 48 × 48 × 30-cm white acrylic glass boxes and monitored throughout the test session (30 min) using a videotracking system (SMART, Panlab S.L., Barcelona, Spain) that recorded the position of the animal every 0.5 s. The same software provided measures of traveled distance, resting time (defined as the time that the animal moved with a speed lower than 5 cm/s), maximum speed, average speed (calculated after elimination of resting time), and time spent in the center of the arena.

#### Elevated Plus Maze (EPM)

Mice were placed in the center of a black acrylic-made maze (2 open arms of 50 × 10 cm and 2 enclosed arms of 50 × 10 × 30 cm elevated to a height of 50 cm above the floor) facing a closed arm, and their behavior was recorded for 5 min with a camera located above the maze. The percentage of time spent and number of entries in the different compartments were assessed.

#### Social Interaction

Mice were placed in an open field with two compartments: one of them containing a mouse (unfamiliar to the experimental subject) while the other was empty. The sociability index was calculated comparing the time spent interacting at each compartment (index = time interacting with the mouse − time interacting with the empty compartment / time interacting with the mouse + time interacting with the empty compartment).

#### Rotarod

Mice were first trained on a Rotarod (Panlab S.L.) at a constant angular speed (15 r.p.m.). They received three trials per day for 5 days. Using this protocol, a steady level of performance was attained in both genotypes. On testing days, the Rotarod was set to increase from 4 to 40 r.p.m. over 300 s and the latency to fall was measured.

#### Marble Burying (MB)

Mice were placed in regular home cage boxes half filled with clean bedding. Upon them, a total number of 12 marble balls were placed symmetrically in two rows along the wall (5 cm separation). Mice were left undisturbed for 15 min and the number of marbled buried (at least 1/3 covered in bedding) was measured.

#### Working Memory in the Y-Maze

In order to test working memory, mice were left to explore a Y-shaped maze made of transparent Plexiglas to promote environmental cues and hippocampal functions. Each trial consisted in three arm visits (triplet). Triplets were considered correct when the mouse visited the three arms without repetition. Triplets were considered incorrect if the mouse visited more than once the same arm. The alternation index was calculated as the number of correct triplets divided by the total amount of possible triplets.

#### Reference Memory in a Water Radial Maze

Mice were habituated to the room for 3 h on the first day. The five following days for 4 trials per day, mice explored a 6-armed water maze of 120 cm in diameter in which one of the arms contained at the end a circular platform of 12 cm in diameter that enabled them to escape from the water. The water was opacified with non-toxic white acrylic paint in order to hide the platform and maintained at 20 ± 1 °C. Three arms contained visual cues of different shape and color, none of which contained the platform. During the visible phase (2 days), the platform changed pseudo-randomly between trials and was cued to assure that the mice succeeded to locate it. During the hidden phase (3 days), the platform was hidden under the water and the mice explored the maze until locating it. If the mouse failed to locate the platform within a minute, it was gently guided towards it. Mice were returned to their home cage after spending 15 s on the platform. On each trial, mice were released from the end of a different arm facing towards the wall. Each time a mouse entered an arm other than the one containing the platform it was counted as an error. For both errors and latency to escape, means of each three trials were calculated. As a result, 3 blocks are represented per day.

#### Porsolt Forced Swimming (FST)

Mice were placed into clear plastic buckets 2/3 filled with 26 °C water. Seconds in immobility were counted. Immobility was considered as the lack of activity other than subtle survival movements and reflexes.

#### Footshock Response and Fear Conditioning Task

Mice were placed in a fear conditioning chamber (Panlab S.L.) and left to explore for 2 min. Afterwards, they were footshocked with an intensity of 0.4. *Freezing* (Panlab S.L.) software was set to consider events longer than 2 s of immobility as freezing, as measured by a piezoelectric accelerometer connected to a transducer. Memory for the context in which the animal received the shock was assessed 24 h later as previously described [17].

### In Vivo Electrophysiology

#### Surgery

Animals were prepared following procedures described elsewhere [18]. Nkx2.1-CBP_KO_ (*n* = 6, 5 males and 1 female) and control (*n* = 5, 4 males and 1 female) littermates of 6 months of age were examined. Under deep anesthesia (ketamine, 35 mg/kg and xylazine, 2 mg/kg, i.p.), mice were implanted with bipolar stimulating electrodes aimed at the right Schaffer collateral-commissural pathway of the dorsal hippocampus (2 mm lateral and 1.5 mm posterior to Bregma; depth from brain surface, 1.0–1.5 mm; Paxinos and Franklin, 2001) and with two recording electrodes aimed at the ipsilateral CA1 area (1.2 mm lateral and 2.2 mm posterior to Bregma; depth from brain surface, 1.0–1.5 mm). Electrodes were made from 50 μm, Teflon-coated, tungsten wire (Advent Research, Eynsham, UK). The final location of the recording electrodes in the CA1 area was determined according to the field potential depth profile evoked by single pulses presented to the Schaffer collateral pathway [18]. A bare silver wire was affixed to the bone as ground. Implanted wires were soldered to a six-pin socket (RS Amidata, Madrid, Spain), which was fixed to the skull with dental cement (see [18] for details).

#### Local Field Potential (LFP) Recordings

LFP recordings were carried out with the behaving animal placed into a small box (9 × 9 cm, with 5-cm-high walls), to avoid overt walking movements. Mice were habituated the box during sessions in which input/output curves curved and thresholds were examined. Analyses in the frequency components of recorded LFPs were carried out for the following frequency bands: delta, 0.5–4 Hz; theta, 4–12 Hz; alpha, 12–15 Hz; beta, 15–30 Hz, and gamma, 25–120 Hz (divided in two sub-bands of 25–50 Hz (low-gamma) and 50–120 Hz (high-gamma). The power spectra and the spectrograms of recorded LFPs were computed according to the fast (FFT) and multi-taper (mTFT) Fourier transforms [19, 20] with home-made programs written in MATLAB platform (version 9.0, R2016a, The MathWorks, Natick, MA, USA) and customized scripts of Chronux software (version 2.11, R2014, website: http://chronux.org/). In all time-frequency maps of statistical significances for the multiple comparisons between spectra: the blue (inferences of type − 1: estimate of spectral power for control mice (*E*_CONTROL_) ≪ estimate of spectral power for CBP-deficient mice (*E*_CBP_)) and brown (inferences of type +1; *E*_CONTROL_ ≫ *E*_CBP_) colors denote significant statistical differences (*P* < 0.05), whereas the green color (inferences of type 0; *E*_CONTROL_ ≈ *E*_CBP_) indicates non-significant difference (*P* > 0.05).

#### Kainate Injection and Recording of Ictal Activity

To study the propensity of WT and KO mice to generate convulsive seizures, animals were injected (i.p.) with the AMPA/kainate receptor agonist kainic acid (8 mg/kg; Sigma, Saint Louis, Missouri, USA) dissolved in 0,1 M phosphate-buffered saline (PBS) pH = 7.4. LFPs were recorded in the hippocampal pyramidal CA1 area for 2 h after the injection. Injected animals were presented with a brief stimulation session (five 200 Hz, 100 ms trains of pulses at a rate of 1/s) 1 h after the injection (see [21] for details). Mice were observed and scored by two independent experimenters to evaluate epilepsy onset.

### RNA Extraction and RT-qPCR Assays

Total RNA from hippocampal tissue of both male and female Nkx2.1-CBP_KO_ (*n* = 3, 2 males and 1 female) and control (*n* = 3, 2 males and 1 female) littermates of 6-month of age was extracted with TRI reagent (Sigma-Aldrich) and reverse transcribed to cDNA. Each independent sample was assayed in duplicate and normalized using GAPDH levels. These procedures have been recently described in [22]. The primers used were: Parv For 5′-3′: CCGGATGAGGTGAAGAAGGT; Parv Rev. 5′-3′: CTGAGGAGAAGCCCTTCAGA; SST For 5′-3′: CTCCGTCAGTTTCTGCAGAAG; SST Rev. 5′-3′: CTGGTTGGGCTCGGACAG; LHX6 For 5′-3′: CTCGAGTGCTCCGTGTGTC; LHX6 Rev. 5′-3′: GGTTCCAAATCGGCTGAAGT; SATB1 For 5′-3′: GTCGCACACCACAGTAAGGA; SATB1 Rev. 5′-3′: TTTGCTGCTGAGACATTTGC; Gapdh For 5′-3′: CTTCACCACCATGGAGAAGGC; Gapdh Rev. 5′-3′: CATGGACTGTGGTCATGAGCC. Prox1 For 5′-3′: CCATGCTGTGTCTCCTGTTT; Prox1 Rev. 5′-3′:

AGGGCATTGGTTTGGATGT; Sp8 For 5′-3′: GAATCCTTGGCTCCCTTCT; Sp8 Rev. 5′-3′:

ACCAGACAACCCATTCATCC; Gfap For 5′-3′: GGACAACTTTGCACAGGACCTC; Gfap Rev. 5′-3′:

TCCAAATCCACACGAGCCA.

### Experimental Design and Statistical Analyses

Statistical analyses and graphs were performed using R statistical computing language in *RStudio* and *SigmaPlot* (Systat Software Inc.). All statistical analyses were two-tailed. Multiple comparisons were computed to test differences between sexes and genotypes. No sex differences were found in tests with a significant main effect of genotype. For pairwise comparison of averages, data were tested for normality using Shapiro’s test and examined with a Q-Q plot. If any of the two samples were significantly non-normal, a non-parametric Mann-Whitney *U*/Wilcoxon rank-sum test was executed instead. If the data from the two samples met the normality criterion, an *F* test was used for equality of variances. When variances between the groups were different, a Welch’s *t* test was employed; otherwise, a standard two-sample *t* test was used. A one-way repeated measures ANOVA was used for comparison of averages among trainings and genotypes. Tukey’s test was performed post hoc to control for multiple comparisons between individual groups. Comparisons among electrocorticographic bands were compared with a two-way repeated measures ANOVA and Holm-Sidak-corrected for multiple comparisons. *P* values were considered significant when *α* < 0.05. In bar graphs, means ± SEM are represented. Weights of animals are described as mean ± standard deviation. *P* values were represented as *** when *α* < 0.001, ** *α* < 0.01, * *α* < 0.05, and # *α* < 0.1. To test effect sizes, Cohen’s *d* was employed when data met parametric assumptions. For data in which one the samples did not have similar variances, Glass’ Δ was employed. If one of the samples was significantly non-normal, Vargha-Delaney A was used to test the effect size.

## Results

### CBP Ablation in MGE Precursors Interferes with Interneuron Development

To produce mice in which CBP is specifically removed from MGE interneuron progenitors, we crossed CBP *floxed* mice [14] with transgenic mice expressing the Cre recombinase under the control of *Nkx2-1* regulatory sequences [15] (Fig. 1A). These mice, referred to as Nkx2.1-CBP_KO_, express the Cre recombinase and lose CBP immunoreactivity within Nkx2.1-expressing domains of the brain, primarily in the most dorsal region of the MGE (Fig. 1B–D). Despite this, Nkx2.1-CBP_KO_ mice were indistinguishable from their control littermates both in terms of weight and appearance (males: Nkx2.1-CBP_KO_ = 29.5 ± 2.86 g, *n* = 9; control = 29 ± 2.23 g, *n* = 7; t_14_ = − 0.19, *p* = 0.85, unpaired *t* test. females: Nkx2.1-CBP_KO_ = 23 ± 2.4 g, *n* = 8; control = 24 ± 3 g, *n* = 9; t_15_ = 0.51, *p* = 0.62). Histological analysis of brain anatomy using Nissl staining did not reveal any gross differences either (Fig. 1E).

We next examined the number of PV+ interneurons, which is the most abundant subtype of telencephalic interneurons [23]. We observed a significant reduction in PV+ interneurons across all hippocampal areas, namely the CA1, CA3, and dentate gyrus (DG) (Fig. 1F–G), as well as in cortical areas (Fig. 1H), but not in other brain regions, like the amygdala, in which interneurons have a different origin (Fig. 1I–J). Next, we focused on the hippocampus for its relevance in cognitive processes and because the sparse number of interneurons in this structure facilitates cell counting. We checked for co-localization between the vesicular glutamate transporter 1 (VGLUT1) and PV (Fig. 1K–L). Since VGLUT1 is expressed by pyramidal cells and PV by interneurons, co-localized pixels primarily corresponded to VGLUT1+ buttons contacting somas and/or dendrites of PV+ cells. Normalization of the signal to correct for the reduced number of PV+ cells still revealed that Nkx2.1-CBP_KO_ mice show a diminished level of co-localization in comparison to control mice (Fig. 1L). This indicates that the lower number of interneurons was not compensated for by enhancing excitatory synaptic connections on the remaining cells.

### Genetic Fate Mapping Confirms that CBP Is Required in MGE Precursors

In order to track the fate of MGE-derived interneurons in wildtype and Nkx2.1-CBP_KO_ mice, we generated mice in which Nkx2.1-driven Cre recombinase expression triggered both the ablation of CBP and the expression of the red fluorescent protein tdTomato (tdTOM) (Fig. 2a). These mice, referred to as Nkx2.1-CBP_KO_/tdTOM, were compared with control Nkx2.1-tdTOM mice, which also expressed red fluorescent interneurons but normal levels of CBP.

Consistent with our previous analyses, KO mice displayed a clear reduction in the number of tdTOM+ cells, indicating again that interneuron development is compromised by CBP ablation in MGE progenitors (Fig. 2b–c). As expected, the nucleus of tdTOM+ neurons in Nkx2.1-CBP_KO_/tdTOM mice did not show CBP immunoreactivity (Fig. 2d). As a control, we also analyzed the *reticular nucleus* of the thalamus, a structure that is rich in PV+ cells that do not originate from the MGE [24]. Interneurons of this region were negative for tdTOM expression in both genotypes (Fig. 2e) and did not show any gross difference in number, thereby highlighting the specificity of our genetic strategy.

### SST+ Interneurons that Escaped Recombination Rebalance Hippocampal Circuits

Interneurons originating in the MGE include two major classes: fast-spiking interneurons that express the calcium buffer PV, and non-fast-spiking interneurons that express the neuropeptide SST. These two classes have a distinct morphology and different functions [3]. Thus, we next evaluated how CBP ablation specifically affects these two populations of MGE-derived interneurons (i.e., tdTOM+).

Consistent with our previous analyses in Nkx2.1-CBP_KO_, mice, the number of PV-expressing interneurons (PV+) was lower in Nkx2.1-CBP_KO_/tdTOM mice than in control Nkx2.1-tdTOM mice in all hippocampal regions (Fig. 3a). Furthermore, counting the number of PV+ neurons that were positive or negative for tdTOM, demonstrated a selective depletion of PV+/tdTOM+ interneurons in KO mice, whereas PV+ cells that were negative for tdTOM (i.e., interneurons that did not originate in the MGE) were not affected (Fig. 3b).

In the case of SST-expressing interneurons (SST+), we obtained similar results: a net reduction in the number of SST+ interneurons in all three hippocampal regions of Nkx2.1-CBP_KO_/tdTOM mice (Fig. 3c), with the SST+/tdTOM+ cells being the ones specifically affected (Fig. 3d). Intriguingly, this quantification revealed a larger number of SST+/tdTOM-cells in the CA1 region of CBP-deficient mice (as compared to control mice) (Fig. 3d, left panel). This hints at the existence of a competition between SST+ interneurons originating in the MGE domain that express the Cre-recombinase and SST+ interneurons originating in other areas. Our analyses indicate that this balance is tipped in favor of the latter population when the production of MGE-originating cells is compromised. This compensation is specific of SST+ interneurons (Fig. 3b did not show this effect in PV+/tdTOM- interneurons), and consistent with the known bias of the dMGE for generating SST+ [15, 25]. Although the increase in the number of non-MGE-derived SST+/tdTOM- interneurons was insufficient to correct for the overall reduction in SST+ neurons in the CA1 field (Fig. 3c), the presence of such cells may constrain the magnitude of electrophysiological and behavioral problems caused by the interneuron shortage.

### CBP Is Essential for the Acquisition and/or Maintenance of Terminal Interneuron Traits

We also quantified the number of cells that were tdTOM+ but did not express the PV or SST markers. Intriguingly, this analysis revealed a larger number of such cells in the CA3 region of Nkx2.1-CBP_KO_/tdTOM mice compared to Nkx2.1-tdTOM mice (Fig. 3e). Interestingly, these tdTOM+/PV-/SST-cells (which, as expected, were negative for CBP expression) were smaller than other cells in the CA3 subfield. However, the few tdTOM+/PV-/SST-cells detected in Nkx2.1-tdTOM mice did not show an abnormal size (Fig. 3f). While an overall reduction in the number of PV+ cells and SST+ cells indicates that CBP is important for the differentiation or survival of MGE-derived interneurons, the abnormal appearance of these tdTOM+/SST-/PV-cells could suggest that CBP is specifically required for the acquisition and/or maintenance of terminal interneuron traits. Alternatively, the abnormal morphology could reflect a pathological state.

Recent studies have shown that the final steps of interneuron maturation and the integration of interneurons into inhibitory circuits require the action of TFs such as Lhx6 and the maturation-promoting factor Satb1 [3, 26, 27]. For this reason, we explored whether the expression of these TFs, in addition to the terminal markers of interneuron maturation, were affected by CBP ablation in MGE neuroprogenitors through RT-qPCR assays. Our experiments revealed a clear decrease in the expression of these TFs, albeit not to the same magnitude and significance as seen for the PV and SST markers (Fig. 3g). Interestingly, we also tested specific markers for the final steps of maturation of caudal ganglionic eminence (CGE)-derived interneurons (e.g., *Prox1*, *Sp8*) and did not observe significant differences in transcript levels. Overall, our analyses show that the impaired development of MGE interneurons caused by CBP ablation is associated with the existence of cells that originated in the MGE but never acquire a terminal interneuron identity. The abundance of such cells in the CA3 subfield might explain why we did not observe a significant reduction in the total number of tdTOM+ cells in this area compared to the CA1 and DG subfields (Fig. 2c).

### Nkx2.1-CBP_KO_ Mice Display Spontaneous Seizures and Altered Local Field Potential (LFP) Recordings During Epileptic Convulsions

**Fig. 4:**
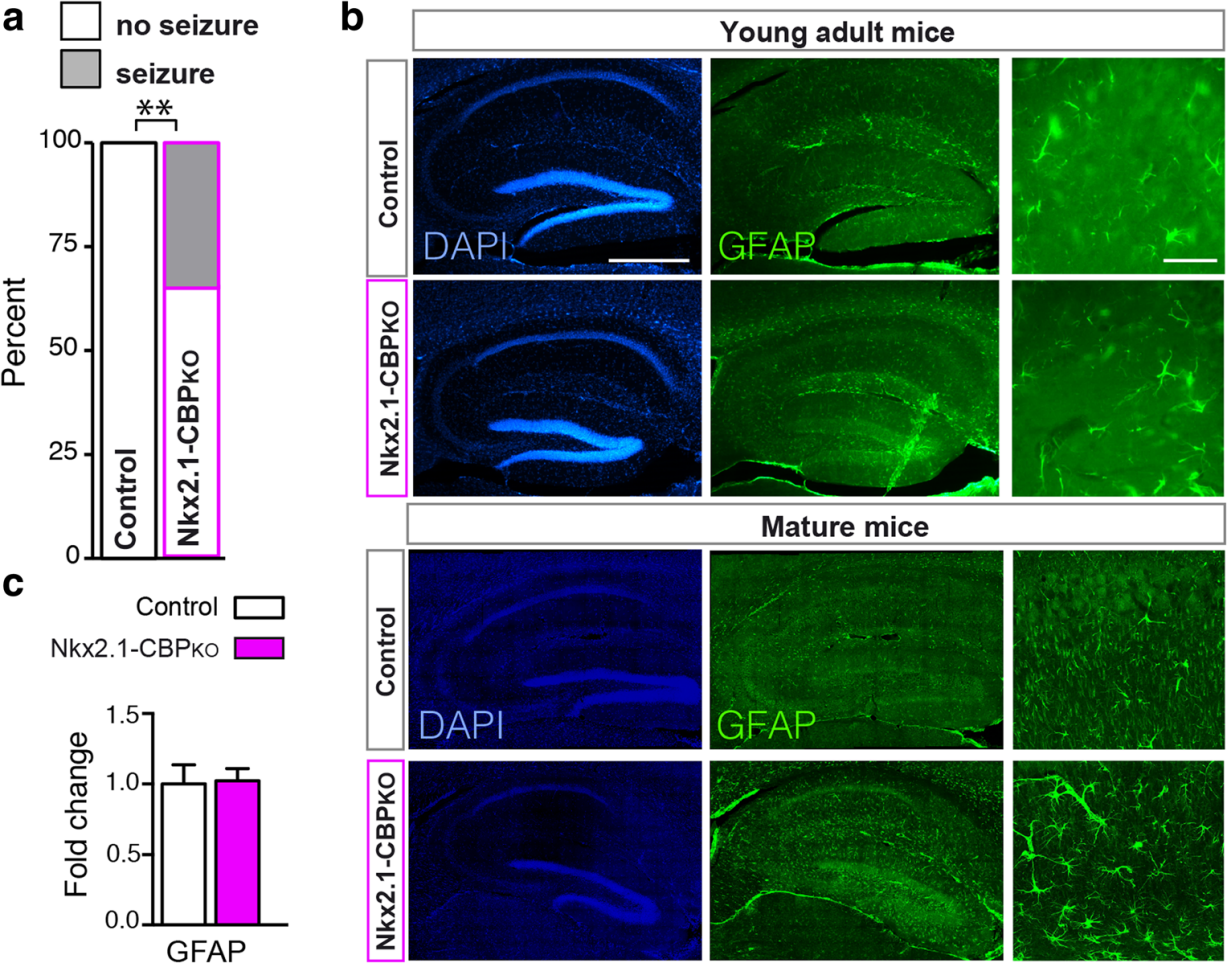
Nkx2.1-CBP_KO_ mice display spontaneous seizures. **a** Incidence of tonic-clonic seizures during behavioral testing. **b** GFAP immunostaining of brain slices from young adult (2 to 3 month-old) and mature (7 to 9 month-old) mice. Only some of the older Nkx2.1-CBP_KO_ mice presented moderate astrogliosis. Scale bars: left panels 500 μm and right panels 50 μm. **P < 0.01. **c** The RT-qPCR assay for the *Gfap* gene using total RNA isolated from the hippocampus of Nkx2.1-CBP_KO_ and control littermates did not show significant difference

During the aforementioned experiments, we observed that some mice displayed spontaneous epileptic seizures during handling. Notably, upon examination of their genotypes, we found that all mice suffering from tonic-clonic convulsions were Nkx2.1-CBP_KO_ (Fig. 4a). In agreement with these episodes, the brain of some of the older Nkx2.1-CBP_KO_ mice used in histological analyses showed a moderate astrogliosis that was not observed in the case of young adult mice (Fig. 4b). RT-qPCR assay for the marker of gliosis *Gfap* did not show significant difference between groups (Fig. 4c).

**Fig. 5:**
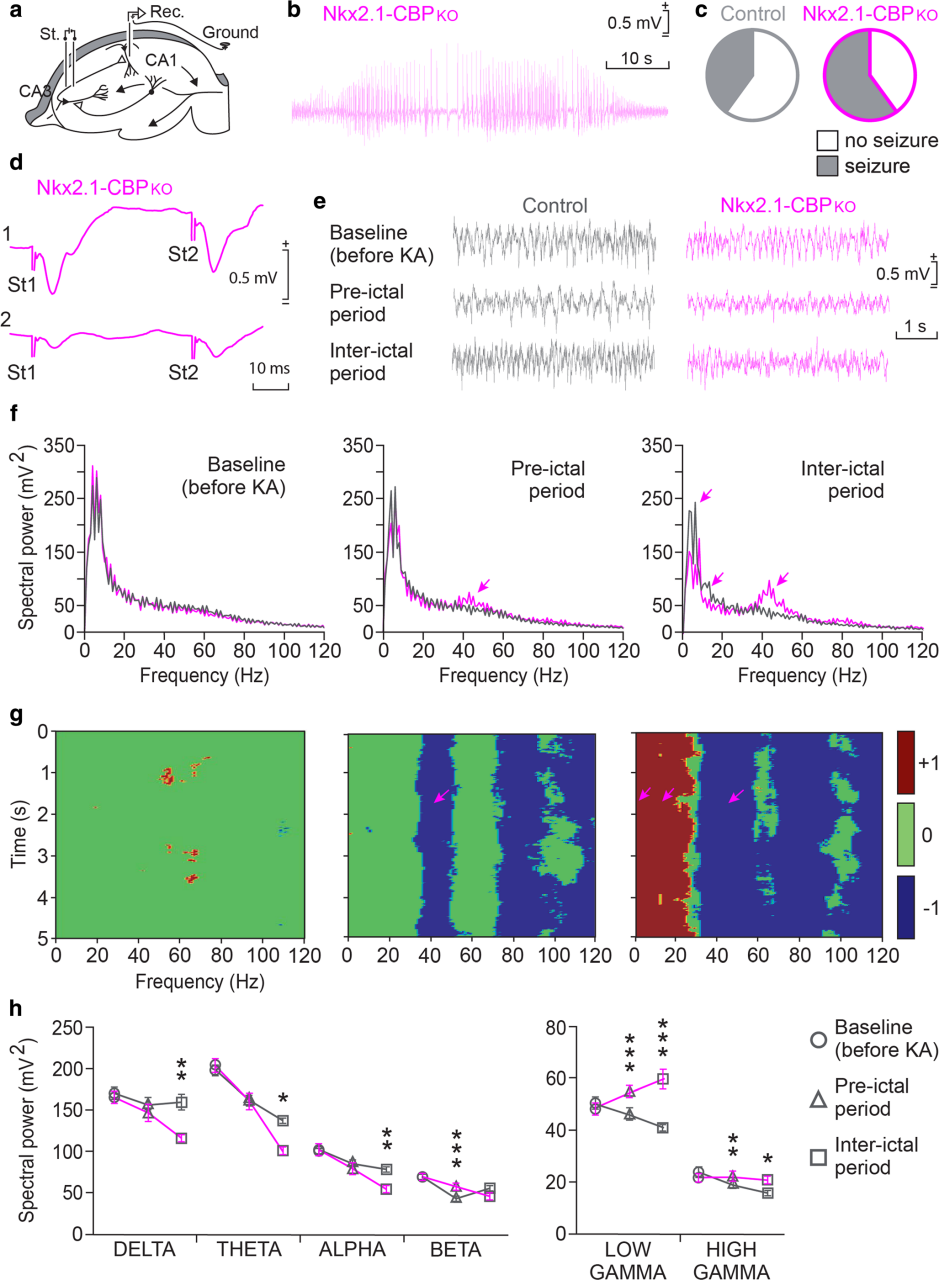
Electrocorticographic characterization of KA-induced seizures in control and Nkx2.1-CBP_KO_ mice. **a** Schematic representation of the stimulating (St.) and recording (Rec.) electrodes implanted in the dorsal hippocampus. Alert behaving mice were recorded for 2 min before and for 1 h after KA injection. Two-minute bins of baseline, pre-ictal, and inter-ictal activities were collected for spectral analysis. **b** A representative example of a hippocampal seizure recorded in an Nkx2.1-CBP_KO_. **c** Pie charts depicting the percentage of mice presenting overt seizures. While 40% of control mice showed epileptic activity, 60% of experimental mice did. **d** Double pulse test carried out in an Nkx2.1-CBP_KO_ mouse before (1) and after (2) an overt seizure. Note the decrease in amplitude of the evoked fEPSP after the seizure. **e** Representative examples of LFP recordings carried out across the different experimental phases and genotypes. **f** Mean power spectra of the LFPs recorded in the CA1 area. Note the presence of sharp peaks in the low gamma band in post-treatment conditions and the smaller spectral power in the theta band during the post-ictal period in Nkx2.1-CBP_KO_ mice (Delta, *F*_(2,546)_ = 9.066, *p* = 0.003, Holm-Sidak-corrected t control vs. Nkx2.1-CBP_KO_ in the inter-ictal period = 2.94, *p* = 0.003; Theta, *F*_(2,546)_ = 2.04, *p* = 0.15, Holm-Sidak-corrected t control vs. Nkx2.1-CBP_KO_ in the inter-ictal period = 2.33, *p* = 0.02; Alpha, *F*_(2,546)_ = 8.16, *p* = 0.004, Holm-Sidak-corrected t control vs. Nkx2.1-CBP_KO_ in the inter-ictal period = 3.05, *p* = 0.002; Beta, *F*_(2,546)_ = 0.04, *p* = 0.84, Holm-Sidak-corrected t control vs. Nkx2.1-CBP_KO_ in the pre-ictal period = 3.28, *p* = 0.001, Low gamma *F*_(2,546)_ = 22.07, *p* = 0.001, Holm-Sidak-corrected t control vs. Nkx2.1-CBP_KO_ in the pre-ictal period = 3.56, *p* = 0.001, Holm-Sidak-corrected t control vs. Nkx2.1-CBP_KO_ in the inter-ictal period = 4.73, *p* = 0.001; High gamma *F*_(2,546)_ = 4.29, *p* = 0.04, Holm-Sidak-corrected t control vs. Nkx2.1-CBP_KO_ in the pre-ictal period = 2.69, *p* = 0.01, Holm-Sidak-corrected t control vs. Nkx2.1-CBP_KO_ in the inter-ictal period = 2.31, *p* = 0.02). **g** Multiple comparisons of LFP spectrograms for the three experimental situations**.** The time-frequency maps of statistical significances (Ps) correspond to the three situations illustrated in (**f**). Note sharp peaks in the low gamma band in post-treatment conditions and a smaller spectral power in the theta band during the post-ictal period for Nkx2.1-CBP_KO_ mice. The color code is illustrated at the right (blue, − 1: *E*_CONTROL_ ≪ *E*_CBP_, *P* < 0.05; red, +1: *E*_CONTROL_ ≫ *E*_CBP_, *P* < 0.05; and green, 0: *E*_CONTROL_ ≈ *E*_CBP_, *P* > 0.05). **h** Spectral analysis of the different frequency bands for baseline recordings (circles), and during pre-ictal (triangles) and inter-ictal (squares) activities after KA injection. Note that a high gamma activity is more evident in CBP-deficient mice (pink lines). *P < 0.05; **P < 0.01; ***P < 0.001

Given the reduced number of PV+ and SST+ interneurons in the hippocampus of Nkx2.1-CBP_KO_, we suspected that their brain activity could be altered. To test this hypothesis, we first chronically implanted intracranial electrodes into the CA1 subfield of the hippocampus of CBP-deficient and control mice and recorded LFPs under baseline and epileptic conditions (Fig. 5a). In order to trigger tonic-clonic seizures, we treated the mice with a low dose of the pro-epileptic drug kainic acid (KA) and evaluated their brain rhythmicity (Fig. 5b–h). While 60% of CBP-deficient mice experienced tonic-clonic seizures, only 40% of control mice did so (Fig. 5c). Surprisingly, despite the reduced number of hippocampal interneurons (both the PV+ and SST+ classes), we did not detect any abnormalities in LFPs recorded before KA treatment (Fig. 5e–f, baseline recordings). After KA treatment on the other hand, clear differences emerged during both the pre-ictal and inter-ictal periods, with the LFPs recorded in CBP-deficient mice having faster (gamma band) components (Fig. 5e–f). As expected, paired pulses before and after a seizure confirmed the depletion of presynaptic vesicles and a reduction in the capacity to respond to repeated stimuli in both genotypes (Fig. 5d). Furthermore, detailed spectral analyses confirmed that there was no difference between genotypes before KA administration (Fig. 5f, left panel). However, after KA administration, gamma frequencies (≈ 40 Hz) were more evident in CBP-deficient mice (Fig. 5f, middle and right panel), while alpha frequencies (≈ 10 Hz) were diminished during the inter-ictal period (Fig. 5f, right panel).

Time-frequency maps revealed that while the two groups of mice presented no statistically significant differences (*P* > 0.05) at baseline conditions (Fig. 5g, left), differences emerged for low- and high-frequency bands following KA administration (Fig. 5g, middle and right graphs). More specifically, LFP activity and its corresponding spectral power at the delta, theta, and alpha bands were significantly (*P* < 0.05) reduced during the ictal period in Nkx2.1-CBP_KO_ mice (Fig. 5h, left graph). In addition, gamma activity was higher in Nkx2.1-CBP_KO_, both in the ictal and non-ictal conditions, with the largest effect being observed in the low gamma band during ictal activity (Fig. 5h, right graph). All together, these results demonstrate that a lack of CBP during interneuron differentiation and maturation compromises brain oscillations in adult hippocampal circuits.

### Nkx2.1-CBP_KO_ Mice Are Hyperactive and Show an Increase in Anxiety and Cognitive Impairments

Interneuron dysfunction has been implicated in a large number of neuropsychiatric conditions, from autism spectrum disorders (ASD) to schizophrenia [4]. We next conducted a series of experiments to test whether Nkx2.1-CBP_KO_ mice show abnormal behaviors associated with dysfunction of inhibitory neurons.

**Fig. 6:**
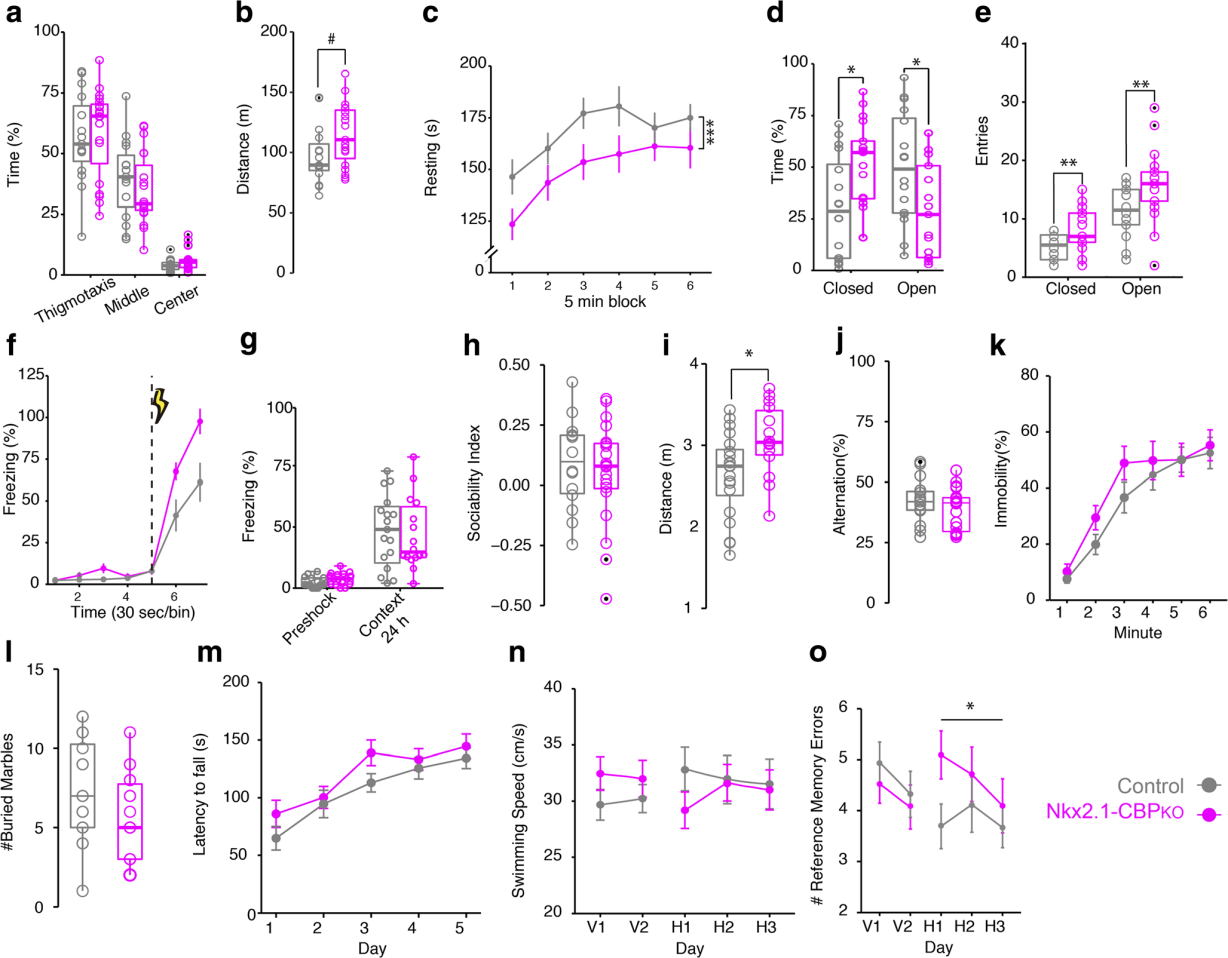
Nkx2.1-CBP_KO_ mice exhibit impaired learning and other behavioral alterations. **a** Time spent in the center and periphery of the arena in an open field task. **b** Total distance run in the open field task. **c** Nkx2.1-CBP_KO_ mice spent less time resting in the open field (*F*_(1,192)_ = 14.01, *p* < 0.001). **d** Time occupancy of the closed and open arms in the EPM (closed arms, *F*_(1,29)_ = 7.06, *p* = 0.01; open arms, *F*_(1,29)_ = 5.82, *p* = 0.02). **e** Number of arms entries in the EPM. **f** Freezing in response to a foot shock. **g** Freezing to the context 24 h after receiving the foot shock. **h** Preference index for interacting with a mouse-containing cylinder or empty cylinder in the three-chamber social interaction task. **i** Total distance traveled in the social interaction task (*F*_(1,29)_ = 6.68, p = 0.01). **j** Working memory in a Y-maze measured as the percentage of turn alternations. **k** Percentage of time spent as immobile in the forced swimming test. **l** Number of marbles buried in the marble-burying test. **m** Latency to fall from the rotarod after various days of training. **n** Swimming speed during training in the radial arm maze. **o** Number of reference memory errors in the radial water maze (hidden phase, *F*_(1,235)_ = 3.76, *p* = 0.05). *P < 0.05; ***P < 0.001

Examination of Nkx2.1-CBP_KO_ in an open field revealed normal occupancy of the different regions in the arena (Fig. 6a). The same mice, however, ran longer distances (Fig. 6b) and displayed a reduced level of resting (Fig. 6c), thereby indicating that they are prone to hyperlocomotive behavior. To test if such behavior was accompanied by an increase in anxiety, we examined the mice in the elevated plus maze (EPM). Nkx2.1-CBP_KO_ stayed for longer amounts of time in the closed arms (Fig. 6d), a behavior which is indicative of increased anxiety. They also showed more arm entries both in the open and closed arms (Fig. 6e), which is consistent with the hyperlocomotion detected in the open filed. Nkx2.1-CBP_KO_ also presented an exacerbated immediate freezing response to a foot shock (Fig. 6f), but no deficit in fear conditioning (Fig. 6g). However, the altered sensitivity to the shock may occlude possible fear memory impairments.

We next explored social behavior in a three-chamber and found that sociability was not affected in Nkx2.1-CBP_KO_ as they spent more time interacting with another mouse than with an empty chamber (Fig. 6h). We also measured locomotor activity during the social interaction test and found that, consistent with the previous observations in the open field, Nkx2.1-CBP_KO_ ran longer distances (Fig. 6i). Some other behaviors associated with interneuron function, such as working memory in a Y-maze (Fig. 6j) and behavioral despair in the Porsolt forced-swimming test (Fig. 6k), were not altered in Nkx2.1-CBP_KO_ (perhaps because of the compensatory presence of non MGE-derived neurons as reported above). Marble-burying behavior (Fig. 6l) and motor coordination in an accelerated rotarod task (Fig. 6m) were also not affected.

Finally, we examined the mice in a reference memory task using a radial-arm water maze. Nkx2.1-CBP_KO_ performed equally well compared to their control littermates in the visible phase of the task and showed the same swimming speed (Fig. 6n). However, they required more time to locate the platform in the hidden phase of the task and made more reference errors (Fig. 6o). This demonstrates that cognitive processes depending on hippocampal function are impaired in Nkx2.1-CBP_KO_.

## Discussion

The correct execution of developmental programs relies on the interaction between environmental cues and a cascade of pre-determined transcriptional programs. Previous loss-of-function studies have identified the sequence of TFs that is responsible for regulating interneuron generation and maturation, and demonstrated that interfering with this sequence leads to excitatory/inhibitory imbalance, seizures and behavioral alterations [3, 26, 27]. For example, the conditional ablation of Nkx2-1 reduced the number of MGE-derived inhibitory cortical interneurons and caused spontaneous seizures and abnormal behaviors [28]. The loss of TFs downstream of Nkx2-1, such as Lhx6 and Satb1, also caused a reduction in the number of interneurons and consequent behavioral abnormalities [29–31]. Some of these TFs are known to interact with chromatin-modifying enzymes, but the specific contribution of these enzymes remains largely unexplored. Here, we identified the transcriptional co-activator and epigenetic enzyme CBP as an important new factor regulating the development and correct integration of mature interneurons into cortical circuits.

Although seminal experiments in vitro previously demonstrated that knocking of CBP in cultured interneuron progenitors interferes with interneuron maturation [13], the in vivo role of CBP in interneuron development had not yet been investigated. Here, we were able to demonstrate that the absence of CBP in MGE progenitors, similarly to what was observed for some of the afore-discussed TFs, caused a reduction in the number of interneurons derived from this region. The cre-driver line used in these experiments utilized the *Nkx2-1* promoter. Previous studies have shown that that promoter starts to be expressed around embryonic day (E) 9 and it is downregulated when neuroprogenitor cells exit the cell cycle [28] and cells migrate out of the MGE, although some subgroups of striatal interneurons maintain its expression at postnatal stages [32, 33]. Since the efficiency of cre-mediated recombination may vary across cells and developmental stages, and there may be additional differences in the stability of preexistent CBP protein after gene ablation, our genetic strategy does not allow a fine dissection between differentiation, migration and maturation deficits. The reduced number of interneurons observed in Nkx2.1-CBP_KO_ mice may result from a combination of these effects. Taken together, our results indicate that CBP is essential for the acquisition and/or maintenance of interneuron identity. It is possible that the TFs involved in interneuron specification are responsible for recruiting CBP to interneuron-specific genes allowing the establishment of the gene program underlying interneuron function. There are remarkable similarities between the phenotypes associated with CBP ablation in MGE progenitors and those in strains deficient for TFs involved in interneuron development. For example, as in the case of *Nkx2-1* conditional knockouts and *Lhx6* hypomorphic mutants, Nkx2.1-CBP_KO_ showed spontaneous seizures, abnormal behavior and altered brain rhythmicity. The similarity between MGE-derived cells after SATB1 [30, 31] and CBP (this study) ablation is particularly striking because in both cases, a number of cells failed to express mature interneuron markers but did not convert to an alternate fate. In addition, in both cases, some regions of the brain were more affected than others. Specifically, we detected a significant increase in the number of SST-/PV-/tdTOM+ cells in the CA3 subfield and a non-significant increase in the same cells in the CA1 subfield (Fig. 3e). This difference might result from the different route and timing of migration of MGE-derived interneurons at the CA3 and CA1 areas [34].

Interestingly, the reduction in the number of mature MGE-derived interneurons did not compromise animal survival. Perhaps, this could be because Nkx2.1-CBP_KO_, in agreement with the findings on *Lhx6* [35] and *Nkx2-1* [28] conditional KOs, presented an increase in the number of hippocampal interneurons that had originated outside the MGE. This could compensate for the reduction in MGE-derived interneurons. In fact, a very recent article investigating Lhx6-deficient mice reported a similar compensatory mechanism for SST+ interneurons, where the postnatal apoptosis of CGE-derived SST interneuron was reduced to compensate for the absence of MGE-derived SST interneurons [36]. These findings suggest that interneurons born in different niches compete with one another to occupy hippocampal circuits. For example, the competing SST+/tdTOM- cells might correspond to a subgroup of SST interneuron that express neuropeptide Y (NPY) recently identified in cell transplantation experiments [37]. Furthermore, in this study, we examined the behavioral consequences of such interneuron production imbalance and compensation, demonstrating that cognitive processes that depend on hippocampal function become impaired. However, we cannot discard that other brain regions displaying a reduced number of interneurons also contribute to the behavioral deficits. Although a recent study has shown that restricted ablation of Nkx2-1 at the embryonic septal neuroepithelium causes learning and memory deficiencies [8], the behavioral consequences of its ablation at the MGE have not been explored. It would be interesting to determine whether restricted ablation of Nkx2-1 and Lhx6 in MGE neuroprogenitors, which cause seizure phenotypes and abnormal electroencephalographic activity [28, 29], also leads to cognitive impairments similar to the ones observed here for Nkx2.1-CBP_KO_ mice.

Studying the role of CBP in interneuron production and maturation has important clinical implications because hemizygous mutations in the human version of the gene (*CREBBP*) cause a severe intellectual disability disorder known as Rubinstein-Taybi syndrome (RSTS; OMIM #180849) that is associated with electroencephalographic anomalies in more than half of the diagnosed individuals. Furthermore, some RSTS patients suffer epilepsy, albeit this is not a common feature of the disease [38, 39]. These clinical observations had a recent correlate in animal models because interneuron production is transiently compromised in CBP hemizygous mice [13] and these mice are prone to audiogenic seizures in the juvenile period (unpublished observations). Intriguingly, this deficit must be compensated for a later time because adult hemizygous mice display a normal number of interneurons and do not show signs of epilepsy [13]. In contrast to the transitory nature of impaired interneuron production in CBP heterozygotes, we showed here that when CBP is selectively ablated in interneuron progenitors, the deficit in interneuron production is permanent and spontaneous seizures continue all the way into adulthood. Moreover, Nkx2.1-CBP_KO_ also showed abnormal LFPs and enhanced gamma activity (highly present during ictal crises in different species) under the influence of the pro-epileptic drug KA. This pattern is consistent with a reduction in the number of PV-expressing (PV+) interneurons, among which fast-spiking basket cells give rise to oscillatory activity in the gamma-frequency range (30–80 Hz). In contrast, beta-frequency oscillations (15–30 Hz) controlled by non-adapting, non-fast-spiking interneurons that express SST were less affected [4]. These deficits are presumably originated during embryonic development (which makes difficult their pharmacological rescue) and might be related to the reported electroencephalographic anomalies in RSTS patients. Since many genes regulating interneuron development and function have been linked to epilepsy, intellectual disability and other pathological perturbations of the excitatory-inhibitory balance [4, 40–42], our findings may also shed light on the etiology of neuropsychiatric disorders associated with a reduced number of interneurons.
